# Therapeutic Protection Against *H. pylori* Infection in Mongolian Gerbils by Oral Immunization With a Tetravalent Epitope-Based Vaccine With Polysaccharide Adjuvant

**DOI:** 10.3389/fimmu.2019.01185

**Published:** 2019-05-28

**Authors:** Le Guo, Dantong Hong, Shue Wang, Fan Zhang, Feng Tang, Tao Wu, Yuankui Chu, Hongpeng Liu, Meng He, Hua Yang, Runting Yin, Kunmei Liu

**Affiliations:** ^1^Ningxia Key Laboratory of Clinical and Pathogenic Microbiology, General Hospital of Ningxia Medical University, Yinchuan, China; ^2^Ningxia Key Laboratory of Cerebrocranial Diseases, Ningxia Medical University, Yinchuan, China; ^3^Department of Medical Laboratory, School of Clinical Medicine, Ningxia Medical University, Yinchuan, China; ^4^Research Center for High Altitude Medicine, Qinghai University, Xining, China; ^5^Clinical Laboratory, People's Hospital of Ningxia Hui Autonomous Region, Yinchuan, China; ^6^Center for Cell Therapy, Affiliated Hospital of Jiangsu University, Zhenjiang, China

**Keywords:** *Helicobacter pylori*, multivalent epitope vaccine, therapeutic vaccine, urease, NAP, CagA, VacA

## Abstract

Urease is an effective target for design of a therapeutic epitope vaccine against *Helicobacter pylori* (*H. pylori*). In our previous studies, an epitope vaccine CTB-UE containing Th and B epitopes from *H. pylori* urease was constructed, and the CTB-UE vaccine could provide therapeutic effect on *H. pylori* infection in mice. However, a multivalent vaccine, combining different antigens participating in different aspects of *H. pylori* colonization and pathogenesis, may be more effective as a therapeutic vaccine than a univalent vaccine targetting urease. Therefore, a multivalent epitope vaccine FVpE, containing Th1-type immune adjuvant NAP, three selected functional fragments from CagA and VacA, and an urease multi-epitope peptide (UE) from CTB-UE, was constructed in this study and expected to obtain better sterilizing immunity than the univalent epitope vaccine CTB-UE. The therapeutic effect of multivalent epitope vaccine FVpE with polysaccharide adjuvant (PA) was evaluated in *H. pylori*-infected Mongolian gerbil model. The results showed that both FvpE and CTB-UE vaccine could induce similar levels of specific antibodies against *H. pylori* urease, and had similar inhibition effect on *H. pylori* urease activity. However, only FVpE could induce high levels of specific antibodies to CagA, VacA, and NAP. In addition, oral therapeutic immunization with FVpE plus PA significantly reduced the number of *H. pylori* colonies in the stomach of Mongolian gerbils compared with oral immunization with CTB-UE plus PA, or FVpE only, and the FVpE vaccine with PA even exhibited sterilizing immunity. The protection of FVpE was related to the mixed CD4^+^ T cell responses and epitope-specific antibodies against various *H. pylori* antigens. These results indicate that a multivalent epitope vaccine targetting various *H. pylori* antigens could be a promising candidate against *H. pylori* infection.

## Introduction

Persistentgastric infection with *Helicobacter pylori* (*H. pylori*) results in several gastric maladies, including gastritis, peptic ulcer disease and gastric cancer (1). Although *H. pylori* infection can be eradicated in a majority of individuals by antibiotic therapy, antibiotic therapy face problems of increasing antibiotic resistance and rising resistance rates (2), and vaccination against *H. pylori* is viewed as a cost-effective alternative to eradication therapy. Generally, preventive vaccines have been widely recognized and accepted. However, given that half of the world's population is already infected with *H. pylori* (3), therapeutic vaccines which could significantly decrease bacterial colonization levels have a wider application prospect. Vigorous immune responses are induced in patients with *H. pylori* infection, but spontaneous removal of *H. pylori* is extremely rare (4). This suggests that *H. pylori* can evade natural immune responses, and allow persistence. Therefore, it may be favorable to achieve sterilizing immunity by triggering the immune responses which are different from nature infection.

Urease (Ure) is considered to be an excellent candidate antigen for therapeutic vaccine against *H. pylori* (5). Several therapeutic subunit vaccines against urease have been developed, but no major breakthrough has been achieved (6, 7). Studies confirmed that urease-specific polyclonal IgG antibodies have no effect on *H. pylori* urease activity when the purified urease was used as the immunogen (8). Therefore, it is speculated that urease multi-epitope vaccine different from the native urease antigen may be effective to achieve sterilizing immunity. In previous study, we constructed a multi-epitope vaccine named CTB-UE containing Th and B epitopes from *H. pylori* urease A (UreA) and B subunits (UreB), and oral therapeutic immunization with CTB-UE significantly decreased bacterial colonization and gastritis (9, 10). In fact, the successful survival and pathogenicity of *H. pylori* in the stomach involves many crucial virulence factors and adhesion factors (11). It is likely that better sterilizing immunity could be achieved by targeting various antigens participating in different aspects of survival and pathogenicity of *H. pylori* than by only targeting urease. It has been reported that a multivalent subunit vaccine including cytotoxin-associated antigen (CagA), vacuolating toxin (VacA), and neutrophil-activating protein (NAP) could reduce *H. pylori* colonization and gastritis in *H. pylori*-infected Beagle dogs and exhibit an antigen-specific cellular response in non-infected volunteers in a phase I study (12, 13). Unfortunately, recent research has confirmed intramuscular immunization with this multivalent subunit vaccine did not confer additional protection against *H. pylori* infection in healthy volunteers after challenge with a CagA-positive strain, despite increased systemic humoral responses to key *H. pylori* antigens (14). Findings from the above studies might suggest that urease should be an indispensable component in multivalent vaccine research, and that immunogenic epitopes should be selected on the basis of their mechanistic interaction with the human immune system. Moreover, an efficient mucosal adjuvant may be needed for therapeutic vaccine against *H. pylori*.

In this study, a multivalent epitope vaccine FVpE, containing Th1-type immune adjuvant NAP, three selected functional fragments (CagA_302−437_, VacA_1−46_, and VacA_332−494_) from CagA and VacA, and an urease multi-epitope peptide (UE) from CTB-UE, was constructed. Moreover, a polysaccharide adjuvant (PA) containing *Lycium barbarum* polysaccharides (LBP) and chitosan was used to assist the FVpE vaccine. Immunological characteristics of FVpE vaccine were analyzed in BALB/c mouse model, and the therapeutic effect of FVpE with PA was evaluated in Mongolian gerbils, in which gastric pathological characteristics of *H. pylori* infection are similar to the manifestations of *H. pylori* infection in humans (15, 16).

## Materials and Methods

### Multivalent Vaccine FVpE Design

The N-terminal domain of CagA (CagA_1−884_) has three structurally distinct domains, named I-III (17). Domain-II of CagA_1−884_ contains 11 antiparallel strands (β1-β5 and β8-β13) forming a specific single-layer β-sheet region (SLB). The first five strands of of SLB (CagA_303−368_) is involved in specific binding of CagA to the β1 integrin (18). The CD4^+^ T cell epitopes binding to MHC class II molecules and linear B cell epitopes of CagA_1−884_ was screened by Epitope Prediction and Analysis Tools (IEDB Analysis Resource, http://tools.iedb.org/main/). The predicted CD4^+^ T cell epitopes with percentile rank ≤ 1 are selected as the candidate epitopes to construct FVpE vaccine. Finally, the fragment (CagA_302−437_), theoretically including the specific binding site (CagA_303−368_) of CagA to the β1 integrin, and CD4^+^ T and B epitope-concentrated region, were selected to construct FVpE vaccine. The mature VacA comprises the p33 and p55 domains. The minimal intracellular vacuolating domain of VacA (VacA_1−422_) corresponds to the entire p33 domain (VacA_1−331_) and about 111 amino acids from the amino-terminal portion of p55 (VacA_332−422_) (19). Deletion of VacA_1−17_ from P33 resulted in the loss of VacA-mediated vacuolating activity, indicating that the amino-terminal region of P33 (VacA_1−17_) is essential for inducing intracellular vacuolation (20). Similarly, the CD4^+^ T cell epitopes and linear B cell epitopes of p33 and p55 domains was screened by Epitope Prediction and Analysis Tools. Finally, the two fragment (VacA_1−46_ and VacA_332−494_), including the amino-terminal portion of P33 and P55 (VacA_1−17_ and VacA_332−422_), and CD4^+^ T and B epitope-concentrated region, were selected to construct FVpE vaccine. The reasonable combination of Th1-type immune adjuvant NAP, the linkers (HM, GS, and KLDPRVPSS), three selected fragments (CagA_302−437_, VacA_1−46_, and VacA_332−494_) and urease multi-epitope peptide (UE) from CTB-UE, were determined by modeling and prediction using RANKPEP, molecular operating environment (MOE) and DNAstar software.

### Construction, Expression, and Purification of FVpE Vaccine

To obtain the recombinant plasmids pET-FVpE, the DNA fragments (CagA_302−437_ and VacA_1−46/332−494_) were synthesized, and the NAP and UE genes were amplified by PCR using *H. pylori* strain 43504 or fusion gene CTB-UE as a template. Four gene fragments (NAP, CagA_302−437_, VacA_1−46/332−494_, and UE) were inserted into pET28a in proper order to construct the recombinant plasmids pET-FVpE. The recombinant plasmids pET-FVpE was transformed into *E. coli* BL21 (DE3). The fusion protein FVpE was purified by Ni^2+^-NTA affinity chromatography according to the manufacturer's instructions.

### The Immunoreactivity of the FVpE Vaccine

The immunoreactivity of FVpE protein was identified by Western blot and ELISA. Purified FVpE or *H. pylori* antigens (NAP, UreA, UreB, VacA, CagA) was applied to 12% SDS-PAGE and transferred onto polyvinylidene difluoride membranes (PVDF, Millipore). The PVDF membrane was incubated with Rabbit anti-*H. pylori* serum (Abace biology, Beijing, China) or Mouse anti-FVpE serum. After washed four times, the membrane was incubated with HRP- Goat Anti-Rabbit IgG (Proteintech) or HRP- Goat Anti-mouse IgG (Proteintech). The positive signals were monitored using Luminescence ECL detection kit (ThermoFisher). Moreover, ELISA plates were incubated overnight at room temperature with 5 μg/mL purified FVpE protein. Serums from *H. pylori*-infected patients or healthy human were collected and diluted 1:100 in PBS. A HRP-conjugated goat anti-human IgG (Jackson ImmunoResearch) was used as the secondary antibody.

### Multipoint Subcutaneous Injection With FVpE

Five to 6 week-old male SPF BALB/c mice were purchased from Experimental Animal Center of Ningxia Medical University. Animal use protocols were approved by the Animal Ethical and Experimental Committee of Ningxia Medical University. Mice were randomly divided into three groups (*n* = 8) and were vaccinated subcutaneously 3 times at 7-day intervals with 100 μg of the purified FVpE, CTB-UE or CTB in complete Freund's adjuvant (1:1) on day 0 and in incomplete Freund's adjuvant (1:1) on days 7 and 14. The pure proteins FVpE, CTB-UE or CTB were used as immunogen in the last booster immunization on days 21. Blood samples were collected at 7 days after the last vaccination to determine antigen-specific levels of IgG or IgA by ELISA.

### Antigen-Specific Antibodies After Subcutaneous Injection

Microtiter plates were incubated overnight at room temperature with 5 μg/mL *H. pylori* antigens (Urease, UreA, UreB, CagA, VacA, or NAP) in 0.05 mol/L carbonate bicarbonate buffer. After three washes with PBS-T, plates were blocked with PBS-T containing 10% non-fat milk for 1 h at 37°C. Two-fold dilutions of sera were added to plates for 2 h at 37°C. After washing, proper dilutions of a HRP-conjugated goat anti-mouse IgG (Jackson ImmunoResearch) or a HRP-conjugated goat anti-mouse IgA (Jackson ImmunoResearch) were added to the plates and incubated for 2 h at 37°C. After further washes, plates were revealed by using TMB as a substrate. Antibody titers were defined as the reciprocal of the last dilution with an OD > 2 times normal mouse serum.

### Neutralizing Antibodies Against *H. pylori* Urease

Mouse anti-FVpE IgG in the anti-serum was purified by Sepharose 4B to which the purified FVpE was attached. The purified anti-FVpE IgG was detected by SDS-PAGE. The effect of anti-FVpE IgG to inhibit urease activity was measured by a urease neutralization test (21, 22). Briefly, the *H. pylori* urease (2 μg in 50 μl) was incubated with various concentrations of the purified specific IgG in microtiter plates. The plates were incubated at 4°C overnight. After addition of 100 μl PBS containing 500 mM urea, 0.02% phenol red, and 0.1 mM dithiothreitol (DTT), the color development was measured at 550 nm at 30 min intervals over a period of 3 h. Percentage inhibition was determined by the following equation: [(activity without antiserum − activity with antiserum)/(activity without antiserum) × 100. Moreover, plates were incubated overnight at room temperature with 10 μg/mL epitope peptide (UreB_321−339_) of the urease active site at 4°C. The anti-UreB_321−339_ antibodies were measured by ELISA assay performed as described above.

### Adherence Inhibition Assay

The effect of mouse anti-FVpE IgG was detected by an adherence inhibition assay (23). Briefly, *H. pylori* suspension (1 × 10^9^ CFUs/ml) was treated with various concentrations of IgG from mice immunized with FVpE, CTB-UE or CTB for 1 h, then added to GES-1 cells, and the plates were incubated for 2 h at 37°C. After washing, the cells were stained with Giemsa. The number of adherent *H. pylori* was counted by an oil-immersion microscope.

### Mucosal Adjuvant Activity of Polysaccharide Adjuvant

The oral vaccines were prepared by using 100 μg of FVpE in 500 μl polysaccharide adjuvant (PA) containing LBP (20 μg/mL) and chitosan (1%, w/w). Closed ileal loop and immunohistochemical analysis were performed to verify whether PA could facilitate the delivery of FVpE to Peyer's patches (24). Briefly, 6 week-old male SPF BALB/c mice were fasted overnight. Then, the mice were sacrificed and the closed ileal loops containing one or two Peyer's patches were prepared. An equivalent amount of FVpE protein with or without PA, was injected into the lumen of ileal loops. After incubation for 1 h, the ileal loops were excised and washed with PBS. The tissue samples were then fixed and freeze-sectioned. The FVpE protein were detected using rabbit anti-*H. pylori* antibody (Abcam, UK) and Goat anti-rabbit IgG conjugated with Alexa Fluor® 647 (Abcam, UK). A well-known M cell specific antibody, anti-Gp2 monoclonal antibody conjugated with Alexa Fluor® 488 (MBL, Japan), was used for localization of M cells in Peyer's patch. Besides, nuclei were stained by DAPI (Sigma, USA).

### Therapeutic Vaccination With FVpE

**Figure 1:**
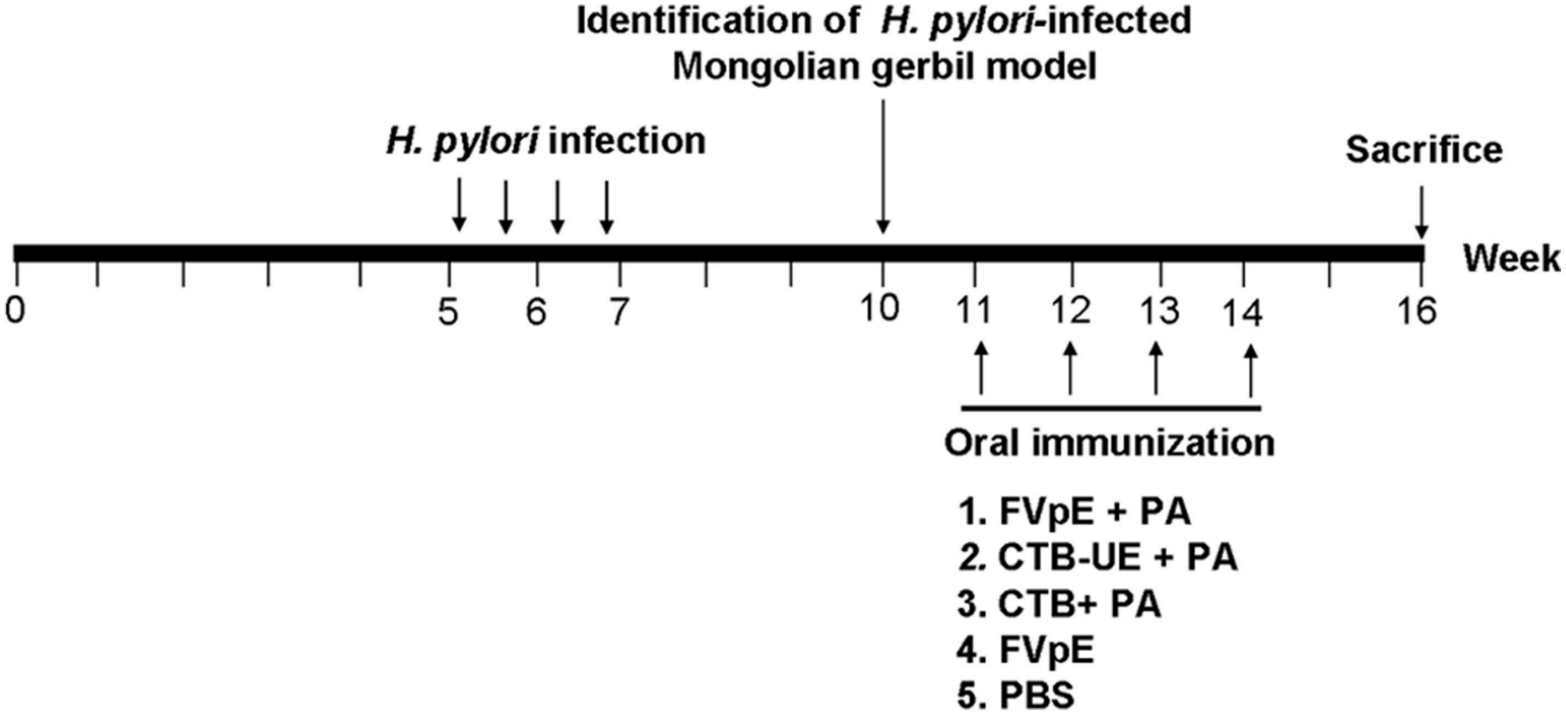
Outline of therapeutic vaccination. Briefly, Mongolian gerbils were orally infected with *H. pylori* SS1 for four times. Three weeks after infection, *H. pylori*-infected Mongolian gerbil model was identificated. The infected Mongolian gerbils were vaccinated intragastrically with FVpE plus PA, CTB-UE plus PA, CTB plus PA, FVpE, or PBS for four times. Mongolian gerbils were sacrificed for various testing items at the 16th week.

Five week-old male Mongolian gerbils were orally infected with *H. pylori* SS1 (10^9^ CFUs) for four times using intubation. Three weeks after infection, three Mongolian gerbils were killed to identificate whether Mongolian gerbils have been successfully infected with *H. pylori*. The infected Mongolian gerbils were randomly divided into three groups (*n* = 10) and were vaccinated intragastrically with 100 μg of FVpE, CTB-UE, or CTB (Absin, Shanghai, China) in 500 μl polysaccharide adjuvant (PA) containing LBP (20 μg/mL) and chitosan (1%, w/w) for 4 times at 1 week intervals. The outline of therapeutic vaccination is shown in Figure 1.

### Assessment of Bacterial Load in Stomach

For assessment of *H pylori* colonization, one stomach fragment was homogenized in 2 ml of PBS, serial Ten-fold dilutions of the homogenate were spread over the surfaces of *H. pylori* selective plate (Qingdao Hope Bio-Technology Co., Ltd.). The plates were incubated for 5–6 days. Colonies were counted to determine the CFU per gram of stomach tissue.

### Urease Activity Determination

one stomach fragment was immediately immersed in 500 μl of the urease substrate. The stomach sample was incubated 4 h at room temperature and the absorbance at 550 nm measured.

### Gastric Histology

One set of biopsies was fixed in buffered 10% formalin solution. Sections 4 μm thick were cut, and stained with hematoxylin-eosin (HE). The gastritis score was determined as described previously (25). Immunohistochemical (IHC) staining was also performed to observe the *H. pylori* infection in the stomach with a rabbit anti-*H. pylori* polyclonal antibody (Linc-Bio, Shanghai, China).

### Specific Antibodies After Therapeutic Vaccination

ELISA plates were incubated overnight at room temperature with 5 μg/mL native *H. pylori* lysates. To detect specific IgG, antisera were collected and diluted 1:800 in PBS. To detect secretory IgA (sIgA), the supernatants from the homogenized stomach tissue or intestinal tissue were collected and diluted 1:5 in PBS. A HRP-conjugated goat anti-mouse IgG (Jackson ImmunoResearch) or a HRP-conjugated goat anti-mouse IgA (Jackson ImmunoResearch) was used as the secondary antibody.

### Adjuvant Activity of NAP in FVpE Vaccine

Lymphocytes were isolated from spleen, and cultured (2 × 10^5^ cells/well) with NAP (5 μg/ml) for 72 h. Then, 10 μL CCK-8 solution was added into plates and incubated for 4 h. The results are expressed as SI. SI = Stimulated cultures (OD_450_)/Negative control cultures (OD_450_). To detect the level of cytokines expression, the supernatants of from splenic lymphocytes were collected 72 h after stimulation with NAP (5 μg/ml) and measured with ELISA (26). Cytokines were quantified by IFN-γ, IL-2, or IL-4 ELISA kits (Shanghai Jiang Lai Biotechnology Co. Ltd., China) following the manufacturer's instructions.

### Specific T Lymphocytes and Cytokine Production

Lymphocytes were isolated from spleen, and cultured with *H. pylori* lysates (5 μg/ml) or urease (5 μg/ml) for 72 h. After stimulation, lymphocyte proliferation was determined by CCK-8. Cytokines were quantified by IFN-γ, IL-4 or IL-17 ELISA kits (Shanghai Jiang Lai Biotechnology Co. Ltd., China) following the manufacturer's instructions.

### Statistical Analyses

Figures represent data from three independent experiments, expressed as mean ± standard deviation (SD), and were analyzed with the GraphPad Prism 5 software using Student's *t*-test. A value of *p* < 0.05 was considered statistically significant. (^*^p < 0.05, ^**^p < 0.01, ^***^p < 0.001; ns, not significant).

## Results

### Multivalent Vaccine FVpE Design and Construction

**Figure 2:**
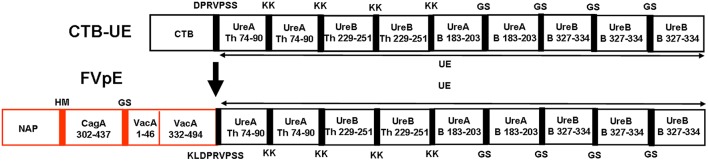
Design of FVpE vaccine. The FVpE vaccine is composed of Th1-type immune adjuvant NAP, three selected fragments (CagA_302−437_, VacA_1−46_ and VacA_332−494_), and Urease multi-epitope peptide (UE) from CTB-UE. Several linkers (HM, GS, and KLDPRVPSS) were designed to separate different fragments.

The fragments (CagA_302−437_, VacA_1−46_, and VacA_332−494_), including known key functional regions, and some known or predicted CD4^+^ T and B cell epitopes, were selected as the components of multivalent vaccine FVpE. The reasonable combination of Th1-type immune adjuvant NAP, the linkers (HM, GS, and KLDPRVPSS), three selected fragments (CagA_302−437_, VacA_1−46_, and VacA_332−494_) and Urease multi-epitope peptide (UE) from CTB-UE, were determined by modeling and prediction using molecular operating environment (MOE), RANKPEP, and DNAstar software. The antigenic structure of FvpE is shown in Figure 2. To obtain the recombinant plasmids pET-FVpE, four gene fragments (NAP, CagA_302−437_, VacA_1−46/332−494_, and UE) were inserted into pET28a in proper order (Supplementary Figure 1A). The recombinant plasmids pET-FVpE were identified by restriction endonuclease digestion using Nco I and Xhol. After digestion, a about 2,000 bp DNA fragment was obtained, which was consistent with the theoretical size of FVpE gene (Supplementary Figure 1B). Moreover, The plasmids pET-FVpE were also confirmed by DNA sequencing (data not shown).

### Expression, Purification, and Immunoreactivity of FVpE

**Figure 3:**
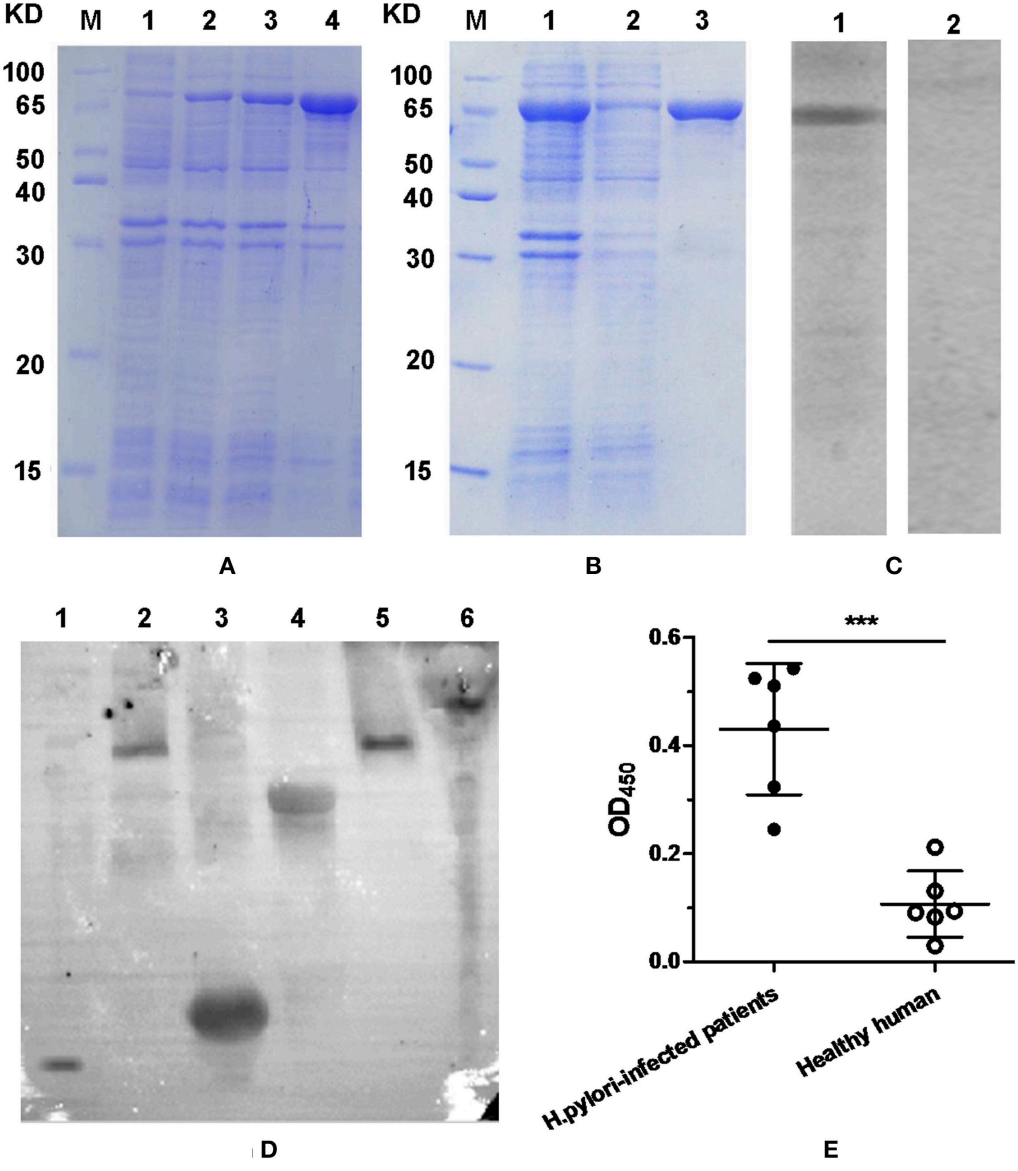
FVpE expression, purification and immunoreactivity. **(A)** Protein expression of FVpE. (Lane M) protein marker, (lanes 1) the soluble proteins of *E. coli* BL21(DE3)/pET-FVpE without induction with IPTG, (lanes 2 and 3) the soluble proteins of *E. coli* BL21(DE3)/pET-FVpE induced by IPTG for 3 or 6 h, (lanes 4) the inclusion bodies of *E. coli* BL21(DE3)/pET-FVpE induced by IPTG for 6 h. **(B)** Protein purification of FVpE. (Lane M) protein marker, (lanes 1) the inclusion bodies of *E. coli* BL21(DE3)/pET-FVpE, (lane 2) the removed non-specific proteins, (lane 3) the purified FVpE proteins. **(C)** The purified FVpE reaction with rabbit anti-*H. pylori* serum. (lane 1) rabbit anti-*H. pylori* serum. (lane 2) normal rabbit serum. **(D)** The *H. pylori* antigens reaction with mouse anti-FVpE serum. NAP (lane 1, 14 KD), VacA (lane 2 and 5, 116KD), UreA (lane 3, 31KD), UreB (lane 4, 64 KD), CagA (lane 6, 116 KD). **(E)** The purified FVpE reaction with serum from *H. pylori*-infected patients. ELISA plates were coated with 5 μg/well of the purified FVpE. The serums from *H. pylori*-infected patients (*n* = 6) and healthy human (*n* = 6) were diluted 100 times. Data are mean ± S.D. ***p < 0.001, ns, not significant.

Result of SDS-PAGE analysis indicated that most of the FVpE protein was in inclusion bodies, and about 82% of total protein (Figure 3A; Lane 4). After purification with Ni^2+^-NTA affinity chromatography, the FVpE protein with over 95% purity was obtained, as analyzed by SDS-PAGE (Figure 3B; Lane 3) and computer scan. Western blot analysis indicated that the FVpE protein could react with rabbit anti-*H. pylori* serum (Figure 3C; Lane 1). However, the FVpE protein could not react with normal rabbit serum (Figure 3C; Lane 2). Furthermore, mouse anti-FVpE polyclonal antibodies could recognize *H. pylori* NAP, UreA, UreB, CagA, and VacA (Figure 3D). The immunoreactivity of FVpE protein was also identified by ELISA. Serums from *H. pylori*-infected patients could react with the purified FVpE protein. However, serums from healthy human could not react with the purified FVpE protein (Figure 3E).

### Specific Antibodies After Subcutaneous Injection

**Figure 4:**
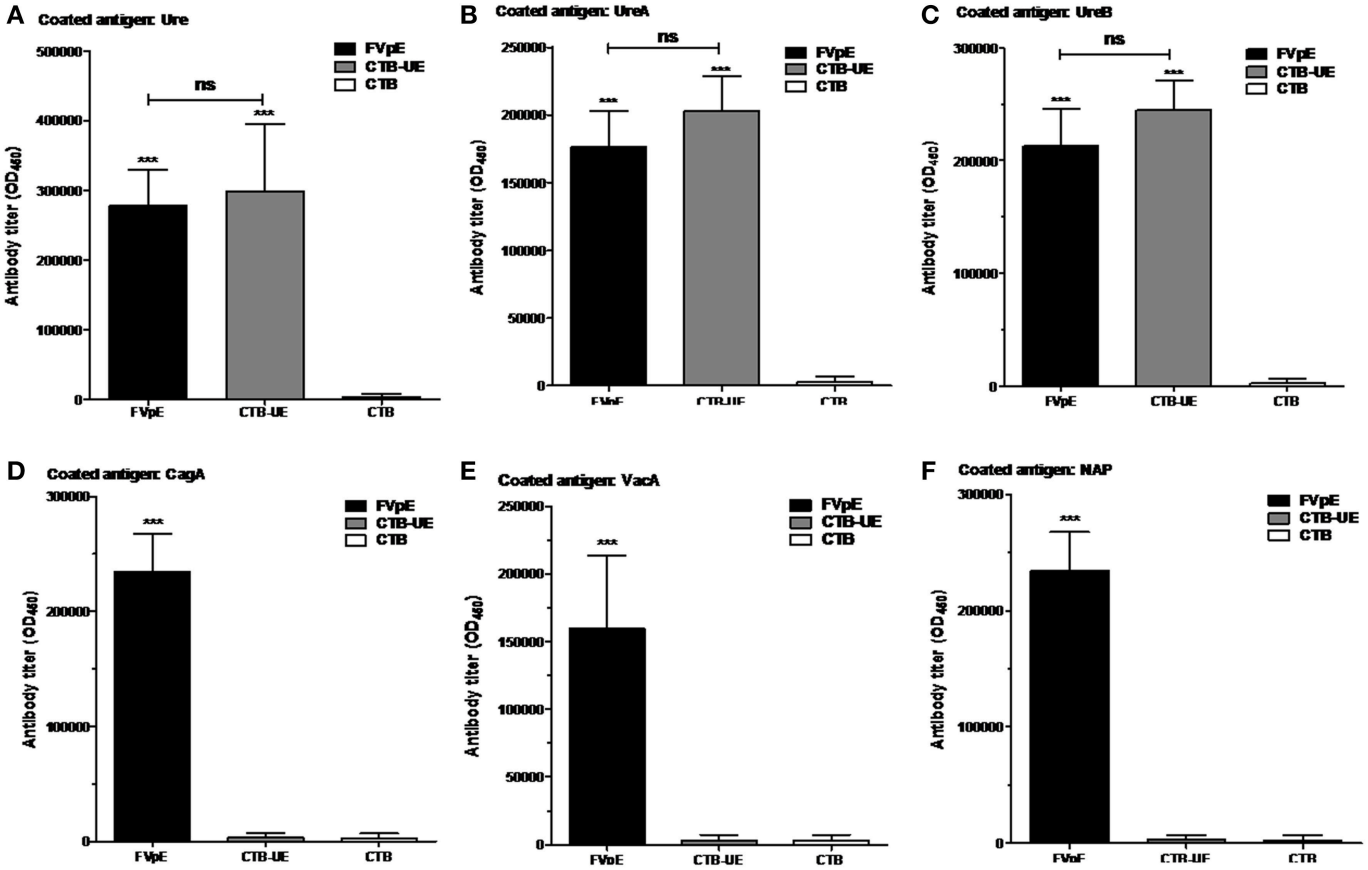
Specific antibodies elicited by subcutaneous injection. BALB/c mice were immunized subcutaneously with FVpE, CTB-UE, or CTB plus Freund's adjuvant. ***p < 0.001, ns, not significant. **(A)** Antibodies against *H. pylori* urease. **(B)** Antibodies against UreA. **(C)** Antibodies against UreB. **(D)** Antibodies against CagA. **(E)** Antibodies against VacA. **(F)** Antibodies against NAP.

The capacity of FVpE to induce serum IgG antibodies against *H. pylori* antigens (Urease, UreA, UreB, CagA, VacA, or NAP) was evaluated by ELISA. The FVpE vaccine could induce similar levels of antibodies specific to Urease (Figure 4A), UreA (Figure 4B), and UreB (Figure 4C) compared with CTB-UE vaccine. Most importantly, the FVpE vaccine could induce high levels of antibodies specific to CagA (Figure 4D), VacA (Figure 4E), and NAP (Figure 4F). However, the CTB-UE vaccine could not induce antibodies specific to CagA, VacA, or NAP.

### Neutralizing Antibodies and Adherence Inhibition Assay

**Figure 5:**
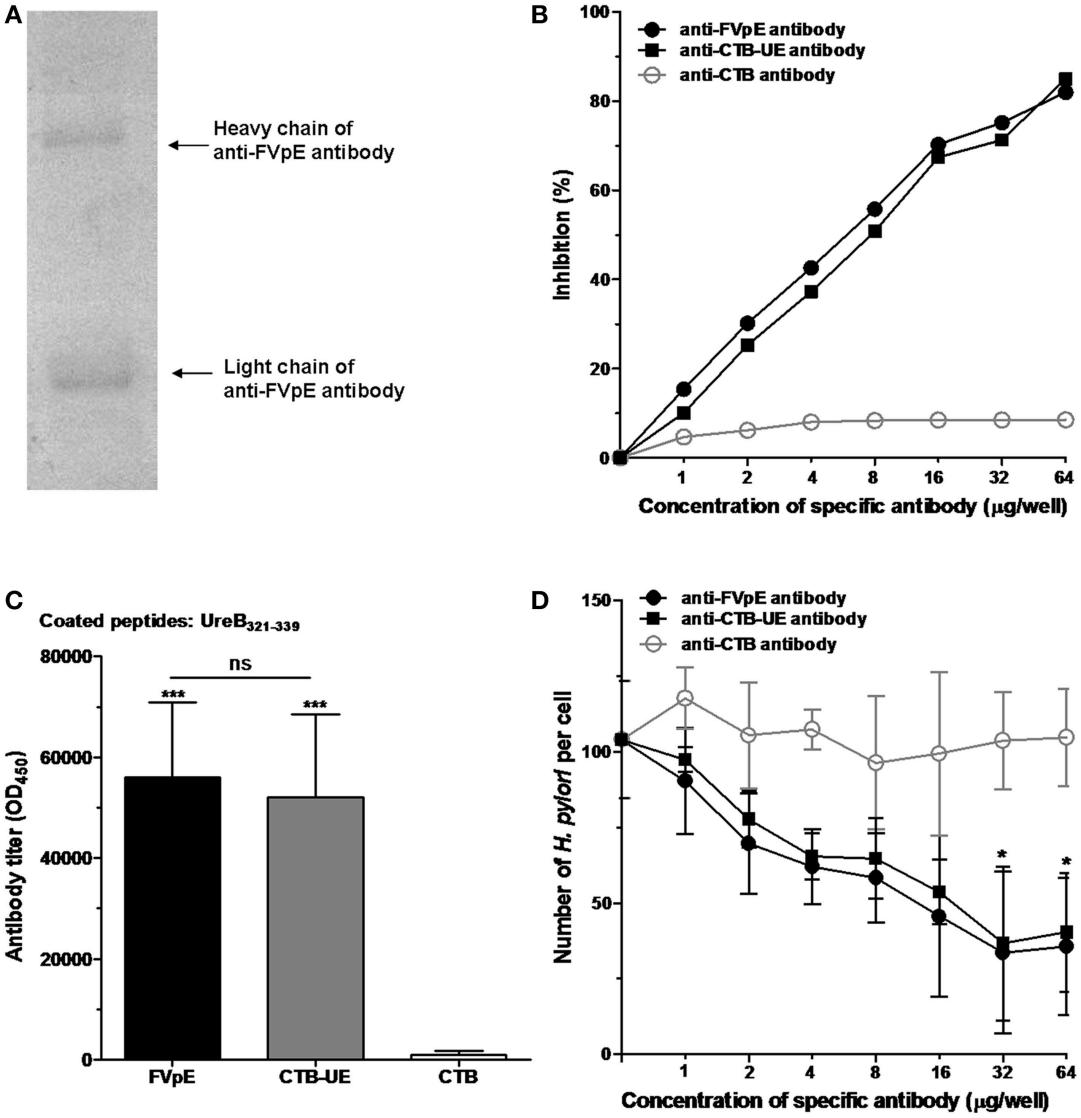
Neutralizing antibodies against urease and adherence inhibition assay. **(A)** Purification of anti-FVpE IgG antibodies. Anti-FVpE antibodies were purified by Sepharose 4B to which the purified FVpE was attached through stable covalent bonds. After purification, anti-FVpE antibodies were detected by SDS-PAGE. **(B)** *H. pylori* urease neutralization test. *H. pylori* urease was pre-incubated with the IgG antibodies (60 μg/well) against FVpE, CTB-UE, or CTB. After addition of phenol red indicator, the color development was measured at 550 nm at 30 min intervals over a period of 3 h. The data are expressed as percentage inhibition. **(C)** Specific antibodies against the UreB_321−339_ epitope peptides of the urease active site. ELISA plates were coated with 1 μg/well of synthetic UreB_321−339_ peptides. ***p < 0.001, ns, not significant. **(D)** Adherence inhibition assay. GES-1 cells were cultured with *H. pylori* pretreated with various concentrations of IgG from mice immunized with FVpE, CTB-UE, or CTB. After stained with Giemsa, the number of adherent *H. pylori* was counted by an oil-immersion microscope. These results were verified in triplicate assays. *p < 0.05 compared with mouse anti-CTB antibody.

Mouse anti-FVpE IgG in the antiserum was purified and identified by SDS-PAGE (Figure 5A). The effect of anti-FVpE IgG to inhibit urease activity was measured by a urease neutralization test. IgG from mice immunized with FVpE or CTB-UE inhibited the urease activity dose-dependently, whereas the IgG from mice immunized with CTB gave no obvious inhibition (Figure 5B). To further examine whether the antibodies induced by FVpE can recognize the enzyme active site of *H. pylori* urease, the UreB_321−339_ epitope peptides of the urease active site were synthesized. Compared with immunization with CTB, immunization with FVpE and CTB-UE significantly increased the levels of IgG antibodies against the UreB_321−339_ peptides (Figure 5C). The multivalent vaccine FVpE could induce neutralizing antibodies against urease, which may be due to the presence of urease multi-epitope peptide (UE) in the FVpE vaccine.

The adherence inhibition assay showed that mouse anti-FVpE IgG and mouse anti-CTB-UE IgG caused a significant dose-dependent reduction in the number of *H. pylori* adhering to GES-1 cells compared with mouse anti-CTB IgG (Figure 5D).

### The Mucosal Adjuvant Effect of Polysaccharide Adjuvant

**Figure 6:**
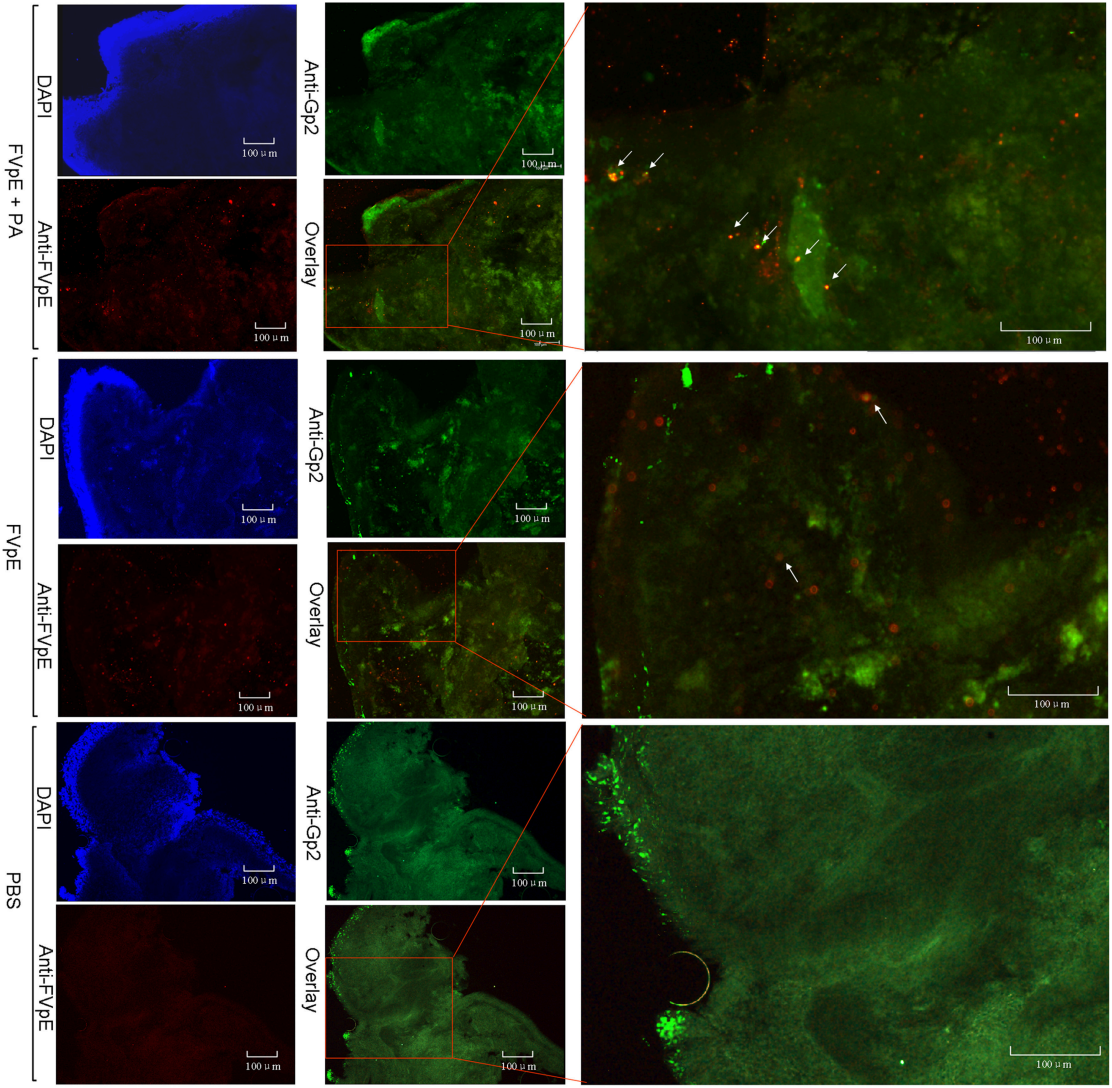
Localization of FVpE in Peyer's patch of mouse small intestine. FVpE plus PA, FVpE, or PBS were injected into the ileal loop and their localization was monitored under fluorescence microscopy. The location of co-localization signals for FVpE binding M cells are indicated by white arrows.

We examined whether PA could be beneficial for the transcytosis of FVpE through M cells. Immunohistochemical analysis demonstrated that the higher efficiency of FVpE to enter from the mucus layer of intestine to inner layer of Peyer's patches in the presence of PA. However, the number of FVpE entered into Peyer's patch was fewer without the presence of PA (Figure 6).

### *H. pylori* Colonization After Therapeutic Immunization

**Figure 7:**
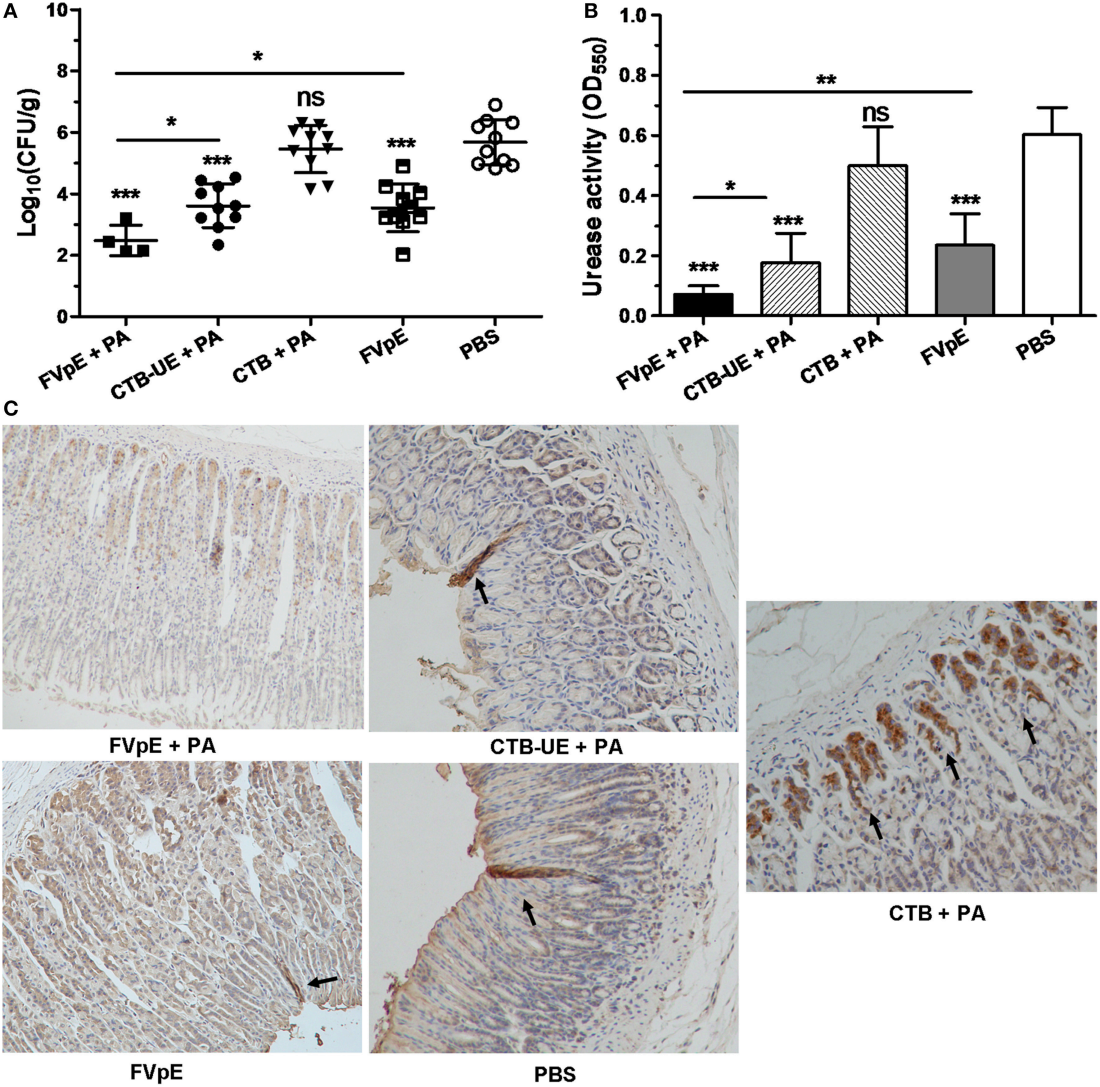
*H. pylori* colonization after oral therapeutic immunization. The *H. pylori*-infected Mongolian gerbils were orally immunized with FVpE plus PA, CTB-UE plus PA, CTB plus PA, FVpE, or PBS. **(A)** Quantitative culture of *H. pylori*. Number of live *H. pylori* per stomach was determined by quantitative culture. Data are mean ± S.D. *p < 0.05, ***p < 0.001. **(B)** *H. pylori* urease activity in the stomach. Urease tests were performed on gastric samples. Data are mean ± S.D. *p < 0.05, **p < 0.01, ***p < 0.001. **(C)** Observation of *H. pylori* by IHC staining. *H. pylori* colonizing in the stomach were marked by black arrows.

To determine whether oral immunization of FVpE vaccine could reduce the bacterial load in the stomach of *H. pylori*-infected Mongolian gerbils, *H. pylori* colonization in the stomach was analyzed by quantitative culture of bacteria, urease activity test and immunohistochemistry (IHC) staining. The result of quantitative culture showed that oral therapeutic immunization with FVpE plus PA induced a significant reduction in the bacterial load in the stomachs of *H. pylori*-infected Mongolian gerbils compared with CTB-UE plus PA or FVpE only (Figure 7A). Six of the ten Mongolian gerbils immunized with FVpE plus PA adjuvant even exhibited sterilizing immunity, i.e., no *H. pylori* could be detected in the stomachs of Mongolian gerbils immunized with FVpE plus PA (clearance rate = 60%). Moreover, oral therapeutic immunization with FVpE plus PA could dramatically reduce urease activity in stomach compared with CTB-UE plus PA or FVpE only (Figure 7B). Besides, Figure 7C show representative IHC results obtained with biopsies from mice immunized with FVpE plus PA, CTB-UE plus PA, CTB plus PA, FVpE, or PBS, respectively.

### Histological Analysis After Therapeutic Immunization

**Figure 8:**
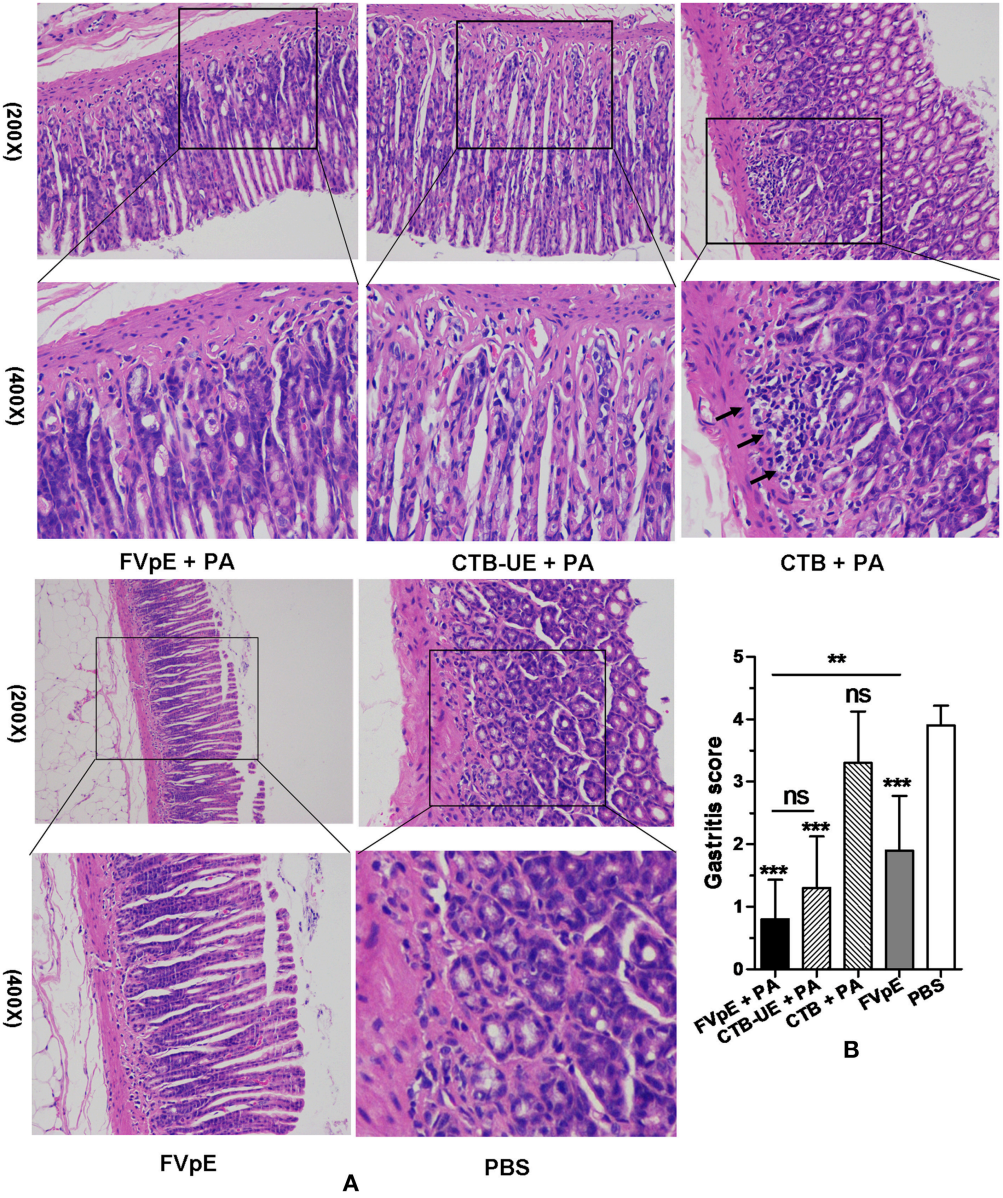
Gastric histology after oral therapeutic immunization (HE stain). The *H. pylori*-infected Mongolian gerbils were orally immunized with FVpE plus PA, CTB-UE plus PA, CTB plus PA, FVpE, or PBS. **(A)** HE staining. *H. pylori*-infected Mongolian gerbils immunized with CTB plus PA or PBS showed severe inflammatory infiltrates. However, *H. pylori*-infected Mongolian gerbils immunized with FVpE plus PA, CTB-UE plus PA, or FVpE only showed mild inflammatory infiltrate. **(B)** Gastritis scores. The inflammatory scores from *H. pylori*-infected Mongolian gerbils immunized with FVpE plus PA, CTB-UE plus PA, or FVpE only was less than that from *H. pylori*-infected Mongolian gerbils immunized with CTB plus PA. **p < 0.01, ***p < 0.001; ns, not significant.

Histology of the gastric biopsies revealed a great many leukocytes and neutrophils in the stomachs from Mongolian gerbils immunized with CTB plus PA or PBS. However, a mild or moderate degree of inflammation was found in the stomachs from Mongolian gerbils immunized with FVpE plus PA, CTB-UE plus PA, or FVpE only. Typical histological images of stomachs from different immunotherapy groups are shown in Figure 8A. The results of histological scoring showed that the scoring grades of Mongolian gerbils immunized with FVpE plus PA were significantly lower than those of Mongolian gerbils immunized with CTB plus PA. However, there was no significant difference in histologic scoring between Mongolian gerbils immunized with FVpE plus PA and Mongolian gerbils immunized with CTB-UE plus PA (Figure 8B). Furthermore, the scoring grades of Mongolian gerbils immunized with FVpE plus PA were significantly lower than those of Mongolian gerbils immunized with FVpE only, indicating that PA can enhance the immunotherapeutic effect of FVpE vaccine.

### *H. pylori*-Specific Humoral Immune Responses After Therapeutic Vaccination

**Figure 9:**
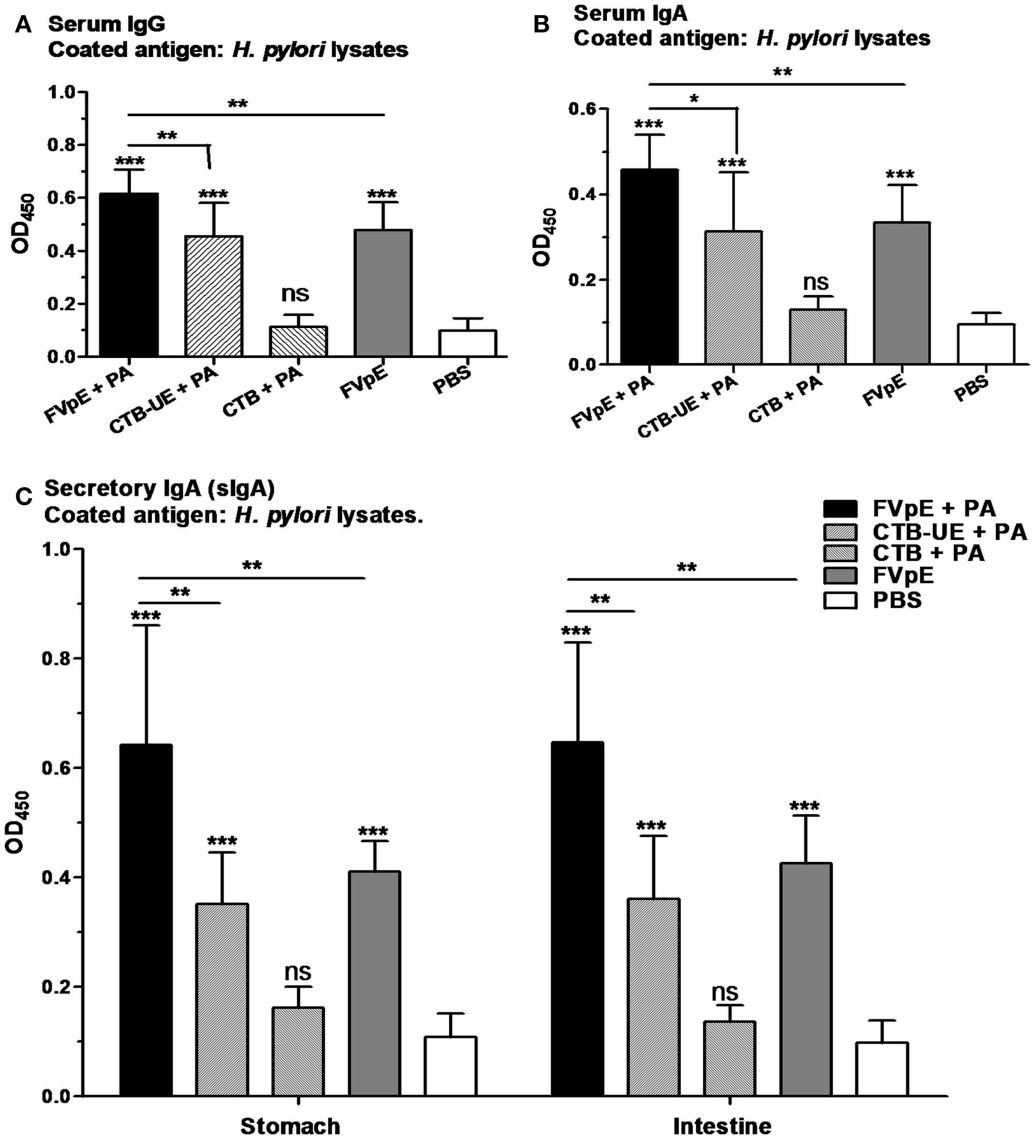
Serum and mucosal antibody responses after oral therapeutic immunization. **(A)** Serum IgG against *H. pylori* lysates. **(B)** Serum IgA against *H. pylori* lysates. **(C)** Gastric and intestinal sIgA against *H. pylori* lysates. Data are mean ± S.D. *p* < 0.05 was considered as statistically significant. *p < 0.05, **p < 0.01, ***p < 0.001, ns, not significant.

The capacity of the FVpE vaccine to induce *H. pylori*-specific serum IgG and IgA antibodies was evaluated by ELISA. Oral immunization with FVpE plus PA, CTB-UE plus PA, or FVpE only significantly increased the levels of serum IgG and IgA against *H. pylori* lysates compared with PBS. However, the FVpE vaccine plus PA could induce higher levels of *H. pylori*-specific serum IgG and IgA antibodies than CTB-UE plus PA (Figures 9A,B), which may be due to NAP, CagA_302−437_ and VacA_1−46_ and VacA_332−494_ components in FVpE vaccine. Furthermore, oral immunization with FVpE plus PA significantly increased the levels of serum IgG and IgA against *H. pylori* lysates compared with FVpE only, suggesting that PA could enhance the immunogenicity of FVpE. Gastric and intestinal mucosal secretory IgA (sIgA) production by each group was also measured. Oral immunization with FVpE plus PA, CTB-UE plus PA, or FVpE only markedly elevated the levels of sIgA against *H. pylori* in gastric mucus and intestinal mucus compared with PBS. Moreover, the FVpE vaccine plus PA could induce higher levels of sIgA against *H. pylori* than CTB-UE plus PA or FVpE only (Figure 9C).

### Th1-Type Adjuvant Effect of NAP in FVpE Vaccine

**Figure 10:**
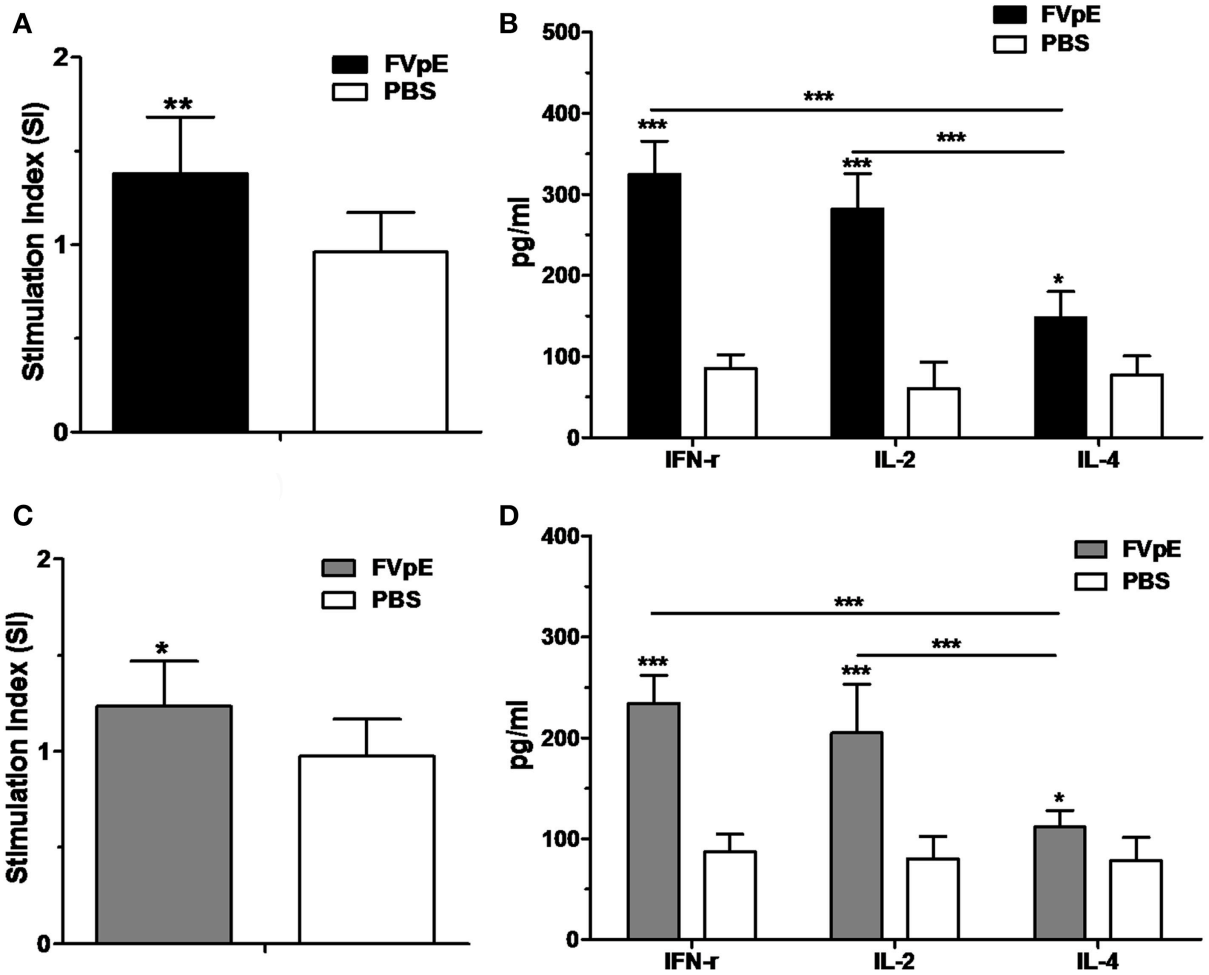
Th1-type adjuvant activity of NAP in FVpE vaccine. **(A,B)** Proliferation of splenic lymphocytes and Th1 cytokine production after stimulation with FVpE. Splenic lymphocytes from *H. pylori*-infected Mongolian gerbils after immunization with FVpE or PBS were stimulated with FVpE for 72 h. Then, the proliferation of splenic lymphocytes and production of cytokines (IFN-γ, IL-2, and IL-4) were detected. *p < 0.05, **p < 0.01, ***p < 0.001. **(C,D)** Proliferation of splenic lymphocytes and Th1 cytokine production after stimulation with NAP. Splenic lymphocytes from *H. pylori*-infected Mongolian gerbils after immunization with FVpE or PBS were stimulated with NAP for 72 h. Then, the proliferation of splenic lymphocytes and production of cytokines (IFN-γ, IL-2, and IL-4) were detected. *p < 0.05, ***p < 0.001.

To determine the Th1-type adjuvant effect of NAP in FVpE vaccine, splenic lymphocytes were stimulated *in vitro* with NAP. Compared with lymphocytes from Mongolian gerbils immunized with PBS, lymphocytes from Mongolian gerbils immunized with FVpE displayed significantly high proliferation after stimulation with FVpE or NAP (Figures 10A,C). Most importantly, levels of Th1 cytokines (IFN-γ and IL-2) were significantly higher than Th1 cytokine (IL-4) in the supernatants of cultured splenocytes from Mongolian gerbils immunized with FVpE after stimulation with FVpE or NAP (Figures 10B,D), indicating that NAP component in FVpE maintained Th1-type adjuvant activity.

### *H. pylori*-Specific Lymphocyte Responses After Therapeutic Vaccination

**Figure 11:**
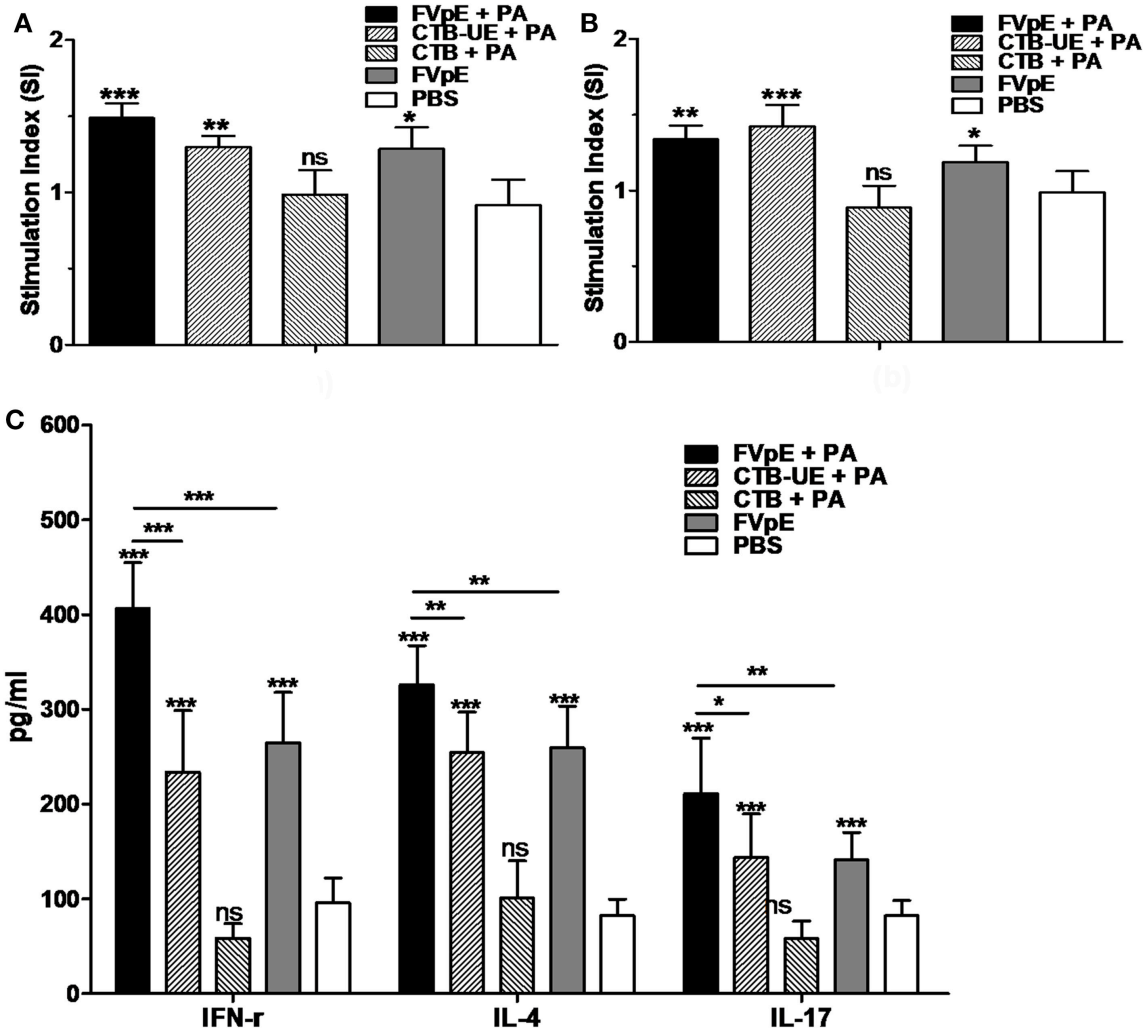
Specific T lymphocytes after oral therapeutic vaccination. **(A,B)** Proliferation of splenic lymphocytes. Splenic lymphocytes from Mongolian gerbils immunized with FVpE plus PA, CTB-UE plus PA, CTB plus PA, FVpE, or PBS were incubated with *H. pylori* lysates **(A)** or *H. pylori* urease **(B)**. Data are mean ± SD. *p < 0.05, **p < 0.01, ***p < 0.001; ns, not significant. **(C)** Cytokine production. Splenic lymphocytes from *H. pylori*-infected Mongolian gerbils after therapeutic immunization with FVpE plus PA, CTB-UE plus PA, CTB plus PA, FVpE, or PBS were stimulated with *H. pylori* lysates for 72 h, and the production of cytokines (IFN-γ, IL-4, and IL-17) was detected by ELSA. Data are mean ± S.D. *p < 0.05, **p < 0.01, ***p < 0.001.

To determine the capacity of FVpE vaccine to induce lymphocyte responses against *H. pylori*, splenic lymphocytes were stimulated *in vitro* with *H. pylori* lysates and urease. Compared with lymphocytes from Mongolian gerbils immunized with PBS, lymphocytes from Mongolian gerbils immunized with FVpE plus PA, CTB-UE plus PA, or FVpE only displayed significantly high proliferation after stimulation with *H. pylori* lysates or urease (Figures 11A,B). Cytokines IFN-γ, IL-4, and IL-17 in the supernatants of cultured splenocytes stimulated with *H. pylori* lysates were detected by ELISA. Stimulation of splenic lymphocytes from Mongolian gerbils immunized with FVpE plus PA, CTB-UE plus PA, or FVpE resulted in significantly higher levels of IL-4, IFN-γ, and IL-17 cytokines than stimulation of cells from PBS-immunized Mongolian gerbils. Moreover, levels of IFN-γ, IL-4, and IL-17 cytokines in the supernatants of splenocytes from Mongolian gerbils immunized FVpE plus PA were significantly higher than that in Mongolian gerbils immunized with CTB-UE plus PA or FVpE only (Figure 11C).

## Discussion

Urease, an important virulence factor and colonization factor for *H. pylori*, is the first *H. pylori* antigen that draw scientist's attention (5). Many studies have showed that urease might be a promising antigen for *H. pylori* vaccine (26, 27). Recently, an oral prophylactic vaccine using urease B subunit (UreB) fused with heat-labile enterotoxin B subunit (LTB) was proven effective, safe and immunogenic in *H. pylori*-naive children and could substantially reduce the incidence of *H. pylori* infection in a phase-3 clinical trial (28). Although there have been many promising results in the field of urease subunit vaccine against *H. pylori*, a therapeutic subunit vaccine using urease to treat or eradicate the infection in human has not been acquired. Studies confirmed that polyclonal IgG antibodies induced by the purified urease have no effect on *H. pylori* urease activity (8), indicating natural urease antigen may have some limitations as a therapeutic vaccine. In addition to *H. pylori* urease, many antigens have been confirmed to be able to trigger immunity against *H. pylori* infection, such as CagA (29), VacA (30), and NAP (31). A univalent vaccine including only one *H. pylori* antigen induces insufficient immunity as a therapeutic vaccine. Multivalent vaccines, combining different antigens participating in different aspects of *H. pylori* colonization and pathogenesis, may be more effective as a therapeutic vaccine. It has been reported a multivalent subunit vaccine containing CagA, VacA, and NAP have significant therapeutic effects on *H. pylori*-infected beagle dogs by intramuscular immunization (12). However, recent research has revealed that this multivalent subunit vaccine did not confer protection against *H. pylori* in healthy volunteers (14). We speculate that natural *H. pylori* antigens may be unsuitable for stimulating sterilizing immunity, and that an efficient mucosal adjuvant may be needed for therapeutic vaccine against *H. pylori*. Therefore, the selected major functional fragments (CagA_302−437_, VacA_1−46_, and VacA_332−494_) and urease multi-epitope peptide (UE) from CTB-UE were used as the components to construct the multivalent epitope vaccine FVpE instead of natural *H. pylori* whole antigens. Moreover, *Lycium barbarum* polysaccharides (LBP) and chitosan have been shown to play a variety of immune-modulatory functions, and are regarded as safe and edible adjuvants (21, 32, 33). We prepared a polysaccharide mucosal adjuvant (PA) containing LBP and chitosan to assist the FVpE vaccine. Experimental results indicated that six of the ten Mongolian gerbils immunized with FVpE plus PA even exhibited sterilizing immunity and the multivalent epitope vaccine FVpE had better therapeutic effect than univalent vaccine CTB-UE.

Generally speaking, the reasons for ineffectual *H. pylori* vaccine development may be insufficient knowledge of protective immune mechanism in *H. pylori* infection. Some earlier studies revealed that humoral immune responses may contribute to protection against *H. pylori* infection (12, 34). However, recent studies suggest that antibodies are not essential for protection (35, 36). In our study, the selected fragments (CagA_302−437_, VacA_1−46_, and VacA_332−494_) and urease multi-epitope peptide (UE) contain many known and predicted B cell epitopes, which were expected to induce epitope-specific antibody response against CagA, VacA, and Urease. Our results indicated that the FVpE vaccine could induce high levels of specific antibodies to various antigens (CagA, VacA, NAP, and Urease), which are key adhesion factors or virulence factors for *H. pylori* colonization and pathogenicity. Beside, the sIgA antibodies against *H. pylori* lysates were found in gastric and intestinal mucus after therapeutic immunization with the FVpE vaccine. Therefore, the FVpE-mediated protective immunity against *H. pylori* infection may be related with humoral immune responses against various antigens, especially epitope-specific antibodies. Most importantly, the multivalent vaccine FVpE could induce neutralizing antibodies against the active site of *H. pylori* urease, which may be due to the presence of urease multi-epitope peptide (UE) in the FVpE vaccine. Isogenic urease-negative mutants of *H. pylori* were incapable of colonizing the gastric mucosa (37). The adherence inhibition assay *in vitro* showed that anti-FVpE IgG could significantly inhibit the adherence of *H. pylori* to human gastric mucosal epithelium GES-1 cells. Furthermore, oral therapeutic immunization with FVpE induced a significant reduction in the bacterial load in the stomachs of *H. pylori*-infected Mongolian gerbils. We speculated that inhibition of urease activity by vaccine-activated neutralizing antibodies can break down the microenvironment of *H. pylori* colonization, and then contribute to clearance of *H. pylori* infection. Current studies suggest that protective immunity against *H. pylori* depends more on CD4^+^ T cell (Th cell) responses than on humoral immune responses (38, 39). Animal models indicate that protective immunity against *H. pylori* involves a strong Th 1 and/or Th17 cell response that promotes a mixed CD4^+^ T lymphocyte response in the gastric mucosa (40, 41). NAP has been identified as not only one of the main *H. pylori* protective antigens, but also an attractive immune adjuvant and inducer of Th1 immunity (42). The whole NAP antigen was selected as not only a protective antigen, but also a Th1-type immune adjuvant in the multivalent vaccine FVpE. Additionally, the selected fragments (CagA_302−437_, VacA_1−46_, VacA_332−494_, and UE) also contained many known and predicted CD4^+^ T cell epitopes, which were expected to induce *H. pylori*-specific CD4^+^ T cell responses. In our study, IFN-γ, IL-17, and IL-4 were all significantly increased in *H. pylori*-infected Mongolian gerbils after therapeutic immunization with the FVpE vaccine, indicating that a mixed CD4^+^ T cell response was induced. The overall evidences suggests that the mixed CD4^+^ T cell response and epitope-specific antibodies against various antigens may contribute to protective immunity against *H. pylori* infection after therapeutic immunization with the FVpE vaccine.

In conclusion, a multivalent epitope vaccine FVpE was constructed by fusing Th1-type immune adjuvant NAP, three major functional fragments from CagA and VacA (CagA_302−437_, VacA_1−46_, and VacA_332−494_), and urease multi-epitope peptide from CTB-UE. The multivalent epitope vaccine FVpE had better therapeutic effect than univalent vaccine CTB-UE with the assistance of polysaccharide adjuvant, which may be related to the mixed CD4^+^ T cell responses and epitope-specific antibodies against various *H. pylori* antigens participating in different aspects of survival and pathogenicity of *H. pylori*. For FVpE as an oral vaccine, it will be necessary to design gastrointestinal delivery system that can effectively enhance the efficacy of oral vaccine. In future studies, we will design a M cell-homing peptide to enhance the mucosal immune effect of FVpE vaccine. In addition, we will express the FVpE protein on the cell surface of lactic acid bacteria by surface display technology and investigate its protective effect. Moreover, clinical studies of the multivalent epitope-based vaccine on humans are expected in the future.

## Ethics Statement

Animals were purchased from Experimental Animal Center of Ningxia Medical University. Animal use protocols were approved by the Animal Ethical and Experimental Committee of Ningxia Medical University.

## Author Contributions

LG, KL, HY, and RY conceived and designed the experiments. DH, SW, MH, FZ, TW, and FT performed the experiments. YC, MH, and HL analyzed the data. LG and RY contributed reagents, materials, and analysis tools. LG, KL, and RY wrote the manuscript.

### Conflict of Interest Statement

The authors declare that the research was conducted in the absence of any commercial or financial relationships that could be construed as a potential conflict of interest.

## Supplementary Material

Supplementary Figure 1 Construction of FVpE vaccine. **(A)** Plasmid pET-FVpE construction. The plasmid pET-FVpE expressing the FVpE protein was obtained by inserting four gene fragments (NAP, CagA_302−437_, VacA_1−46/332−494_ and UE) into pET28a vecter. **(B)** Plasmid pET-FVpE identification. 1, DNA marker; 2, the plasmid pET-FVpE digested by Nco I and Xhol; 3, the plasmid pET-FVpE.
