# Supplementary figures and images for: Therapeutic Protection Against H. pylori Infection in Mongolian Gerbils by Oral Immunization With a Tetravalent Epitope-Based Vaccine With Polysaccharide Adjuvant
Source: Front Immunol. 2019 May 28;10:1185. doi: 10.3389/fimmu.2019.01185 (PMC6546824; doi:10.3389/fimmu.2019.01185)

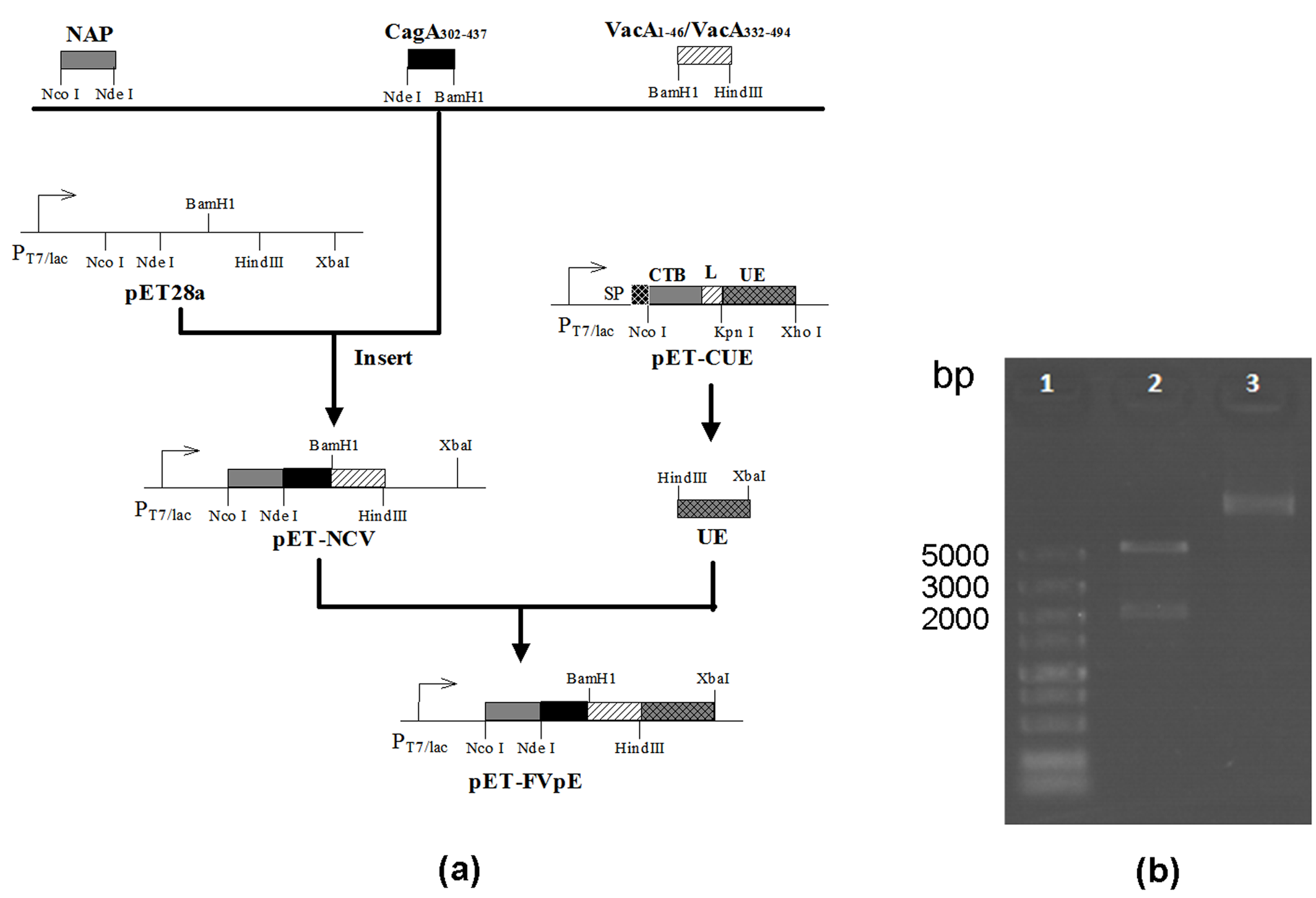

Supplement: Supplementary Figure 1 — Construction of FVpE vaccine. (A) Plasmid pET-FVpE construction. The plasmid pET-FVpE expressing the FVpE protein was obtained by inserting four gene fragments (NAP, CagA302−437, VacA1−46/332−494 and UE) into pET28a vecter. (B) Plasmid pET-FVpE identification. 1, DNA marker; 2, the plasmid pET-FVpE digested by Nco I and Xhol; 3, the plasmid pET-FVpE. [file Image_1.tif]
